# Robot-assisted reaching exercise promotes arm movement recovery in chronic hemiparetic stroke: a randomized controlled pilot study

**DOI:** 10.1186/1743-0003-3-12

**Published:** 2006-06-21

**Authors:** Leonard E Kahn, Michele L Zygman, W Zev Rymer, David J Reinkensmeyer

**Affiliations:** 1 Sensory Motor Performance Program, Rehabilitation Institute of Chicago, Chicago, Illinois, USA; 2 Department of Biomedical Engineering, Northwestern University, Evanston, Illinois, USA; 3 Department of Physical Medicine and Rehabilitation, Northwestern University Feinberg School of Medicine, Chicago, Illinois, USA; 4 Department of Mechanical and Aerospace Engineering, Center for Biomedical Engineering, University of California, Irvine, California, USA

## Abstract

**Background and purpose:**

Providing active assistance to complete desired arm movements is a common technique in upper extremity rehabilitation after stroke. Such active assistance may improve recovery by affecting somatosensory input, motor planning, spasticity or soft tissue properties, but it is labor intensive and has not been validated in controlled trials. The purpose of this study was to investigate the effects of robotically administered active-assistive exercise and compare those with free reaching voluntary exercise in improving arm movement ability after chronic stroke.

**Methods:**

Nineteen individuals at least one year post-stroke were randomized into one of two groups. One group performed 24 sessions of active-assistive reaching exercise with a simple robotic device, while a second group performed a task-matched amount of unassisted reaching. The main outcome measures were range and speed of supported arm movement, range, straightness and smoothness of unsupported reaching, and the Rancho Los Amigos Functional Test of Upper Extremity Function.

**Results and discussion:**

There were significant improvements with training for range of motion and velocity of supported reaching, straightness of unsupported reaching, and functional movement ability. These improvements were not significantly different between the two training groups. The group that performed unassisted reaching exercise improved the smoothness of their reaching movements more than the robot-assisted group.

**Conclusion:**

Improvements with both forms of exercise confirmed that repeated, task-related voluntary activation of the damaged motor system is a key stimulus to motor recovery following chronic stroke. Robotically assisting in reaching successfully improved arm movement ability, although it did not provide any detectable, additional value beyond the movement practice that occurred concurrently with it. The inability to detect any additional value of robot-assisted reaching may have been due to this pilot study's limited sample size, the specific diagnoses of the participants, or the inclusion of only individuals with chronic stroke.

## Background

Given the broad range of therapy approaches currently practiced in clinics, therapists face the difficult task of selecting optimal rehabilitation interventions for hemiparetic stroke survivors. One of the most basic decisions is whether or not to provide mechanical assistance during training movements for patients who are too weak or uncoordinated to move successfully by themselves. "Active-assist" exercise is employed in many clinical practices and is consistent with task-specific exercise advocated in standard rehabilitation textbooks (e.g. Carr and Shepherd [1]). In this approach, a patient will attempt to make a volitional movement while the therapist provides some form of support for the limb and mechanical assistance to complete the desired movement. Different forms of active-assist have been implemented with rehabilitation equipment ranging from simple overhead slings and arm skateboards to sophisticated robotic devices [2,3].

Two arguments support the use of active-assist therapies. First, helping a patient complete an arm movement stretches muscles and soft tissue, which may be helpful in reducing spasticity [4-6] and preventing contracture [7]. Second, helping a weakened patient complete a movement through a normal range of motion introduces novel somatosensory input that otherwise would not be experienced. This enhanced somatosensory input might help drive neural reorganization, and enhance movement planning. For example, purely passive movement activates some cortical areas that are also active during voluntary movement [8-10]. Passive training can also stimulate long term plasticity in both sensory and motor cortices of healthy subjects [10]. Thus, active-assist exercise might be expected to combine the known benefits of repetitive movement exercise [11,12] with the possible benefits of stretching and enhanced somatosensory input.

Robotic devices have recently been introduced to the rehabilitation arena as tools to facilitate repetitive practice of limb movement, specifically in the upper extremity. The first among these, the MIT-MANUS, confirmed that performance of planar reaching movements with assistance from a mechanized device was an effective supplement to conventional therapy in a subacute population [13]. The device has since been used by over 120 individuals in both the subacute and chronic stages of hemiparetic stroke [14,15] and has been made commercially available as InMotion2. Lum and colleagues [3] also demonstrated that combinations of unimanual and bimanual active and passive whole arm exercises in 3-D with the Mirror Image Movement Enabler (MIME) resulted in greater functional improvements than matched doses of Neurodevelopmental Treatment (NDT). Two other robots introduced in Europe further supported the potential benefits of robot-mediated therapy. The GENTLE/S device is a modification of a commercial 3-D robot that yielded greater functional improvement rates than overhead sling training [16]. A simpler device developed by Hesse and colleagues [17] utilized a single motor for each limb and changes in configuration allowed users to practice bilateral wrist flexion/extension and forearm pronation/supination. They noted decreased spasticity and increased motor function in many participants after training.

These studies have collectively demonstrated that both acute and chronic stroke survivors who receive a greater amount of upper limb exercise, provided by a robotic device, recover more movement ability. The baseline and long-term evaluations from many of these studies also have helped to establish a trend of minimally changing arm function over time in individuals who are more than six months post-injury and not receiving any sort of intervention (for a more detailed review please see [18]). As seen in these studies' outcomes, addition of a robotic intervention in a chronic stroke population revealed the continuing potential for functional gains, further justifying the investigation of such therapies long after injury. However, it remains unclear whether the extra exercise dosage of movement practice or the mechanical nature of the therapeutic interaction with the devices (i.e. the active assistance) caused the improved motor outcomes in these studies.

We hypothesized that active-assist exercise with a robotic device would promote upper extremity functional recovery in persons with chronic hemiparesis. We further hypothesized that these improvements in function would be superior to those achievable through simple voluntary repetitive movement training. Accordingly, the purpose of this study was to compare robotic, active-assist exercises with repetitive volitional reaching movements in promoting arm movement recovery in stroke patients with chronic hemiparesis. One randomized group of subjects practiced a fixed number of active-assist reaching movements over a two month period, while a second group practiced an equal number of reaching movements without assistance.

## Methods

### Subjects

Nineteen stroke survivors with hemiparesis resulting from unilateral stroke at least one year previously were recruited from the outpatient population at the Rehabilitation Institute of Chicago and from a participant database (Table 1). A power analysis indicated that with ten subjects in each group there would be a 70% chance of detecting an improvement in the robot-trained group that was at least one standard deviation larger than the improvement in the free-reaching group at the 0.05 significance level [19], a difference that we estimated would be clinically significant (i.e. effect size index = 1). This calculation was based on the assumption that the magnitude of the difference in movement changes between the groups would equal the standard deviation of the population, an assumption consistent with previous studies of robot-assisted movement training [3,13].

**Table 1:** Descriptive data on subject population

Subject group		N	Mean age (SD) [years]	Sex [M/F]	Mean time post-stroke (SD) [months]	Lesion hemisphere [L/R]	CM score at enrollment (SD)
	Severely impaired	6					
Robot trained			55.6 (12.2)	4/6	75.8 (45.5)	5/5	3.5 (0.9)
	Moderately impaired	4					

	Severely impaired	6					
Free reaching trained			55.9 (12.3)	7/2	103.1 (48.2)	6/3	3.2 (1.0)
	Moderately impaired	3					

	Robot trained	6					
Severely impaired			55.9 (10.5)	8/4	99.2 (47.9)	9/3	2.7 (0.5)
	Free reaching trained	6					

	Robot trained	4					
Moderately impaired			55.4 (14.9)	3/4	71.3 (45.0)	5/2	4.3 (0.5)
	Free reaching trained	3					

Subjects experienced varying levels of exercise activity outside of the study, but all had ceased formal physical and occupational therapy, and were instructed not to change their routines during the study. Exclusionary criteria were: difficulty understanding the experimental tasks, cerebellar lesions, hemispatial neglect, severe sensory loss, shoulder pain, and severe contracture or muscle wasting. Twelve subjects with severe impairment (described in the Statistical Analysis subsection) were recruited along with seven subjects with moderate impairment. The impairment level classification was of secondary interest in this study and random sampling resulted in an uneven distribution between the two impairment groups. All procedures were approved by the Northwestern University Institutional Review Board in accordance with the Helsinki Declaration, and subjects provided informed consent.

### Procedure

Participants were stratified by their scores on the arm section of the Chedoke-McMaster (CM) Stroke Assessment Scale. A CM score of 1 represents complete paralysis, and a score of 2 indicates a trace level of elbow or shoulder movement. Scores 3 to 6 mark progressively improved range, coordination, and speed of movement, with a score of 7 indicating an unimpaired arm. The CM scale has high inter- and intra-rater reliability as well as strong correlation with score on the Fugl-Meyer scale because it measures similar movements [20]. Only subjects with a score between 2 and 5 were included, as this range of patients appeared to have the highest potential to benefit from the two modes of training used here (i.e they were able to move to at least some degree, but their movement was distinctly impaired).

**Figure 1:**
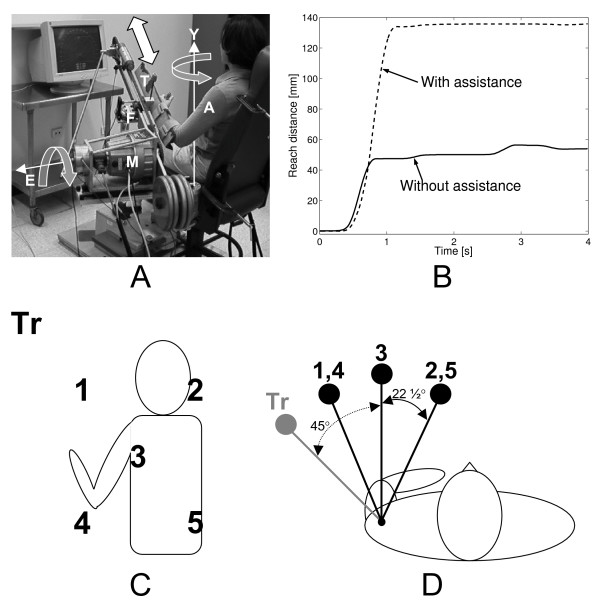
**Description of experimental setup**. (a) Photograph of the Assisted Rehabilitation and Measurement (ARM) Guide. A motor (M) actuates a hand piece and forearm trough (T) attached to a user's arm (A) back and forth along a linear track. A six-axis force sensor (F) measures the interaction forces between the user and the device. The ARM Guide can be oriented on a vertical elevation axis (E) and horizontally on a yaw axis (Y). (b) Example of an unassisted (solid line) and motor-assisted (dashed-line) reach by a hemiparetic subject along the ARM Guide. (c,d) Horizontal and vertical arrangements of the targets used for free reaching assessment, free reaching therapy, and robot-based therapy.

After initial stratification, subjects were randomly assigned to one of two experiment groups. One group engaged in robot-guided active-assist training, and the second in "free reaching training" that involved unconstrained, unassisted repetitive voluntary reaching. Both groups completed an eight-week therapy program involving a total of 24 45-minute exercise sessions. Both groups began each training session by performing eight voluntary reaches along the mechanical device used for training without assistance from the motor, in order to gauge maximum voluntary range and velocity throughout the program. A single exercise session consisted of 10 reaches to each of five targets at different locations in the workspace (1 thru 5 in Figure 1C,D) for a total of 50 movements for both training groups.

#### Active-assist training

Active assistance to movement was provided using a simple robotic device (the Assisted Rehabilitation and Measurement Guide, ARM Guide) that uses a motor and chain drive to move the user's hand along a linear rail in a manner similar to a trombone slide (Figure 1A) [21]. The linear rail can be oriented at different yaw and pitch angles to allow reaching to different workspace regions. The device is statically counterbalanced so that it does not gravitationally load the arm. The hand piece consists of a trough for the forearm and a 2 cm diameter cylinder placed in the palm of the user's hand. Regardless of whether the user was capable of grasping the cylinder, two elastic straps around the proximal and distal forearm fixed this segment to the trough (Figure 1A) and ensured coupling of the user to the device. A strap across the sternum and over the shoulders minimized trunk movement during the reaching tasks. More details of the device design can be found in earlier publications [22-24].

Subjects randomized to the robotic training group performed reaching movements under their own power and control while receiving active assistance from the device. The targeted normative movements were along a straight line path (linear rail of the ARM Guide) and followed the smooth translation profile with a bell-shaped velocity typical of unimpaired reaching movements [25-28] (Figure 1B). The active assistance algorithm remained idle until a subject initiated movement through at least 1 cm along the track in the outward direction toward the target. After the user advanced the hand piece by 1 cm, the controller would monitor the velocity and position trajectories to detect deviations from the targeted movement in real time. To emphasize the importance of subjects moving under their own efforts, a one centimeter deadband in the position trajectory allowed a subject to be within a small margin of error along the planned path before the motor would provide assistance. Outside of this deadband the motor assisted the subject in maintaining the correct trajectory with a force proportional to a weighted sum of the position and velocity errors.

All reaching movements were practiced over the subject's entire supported passive range of motion (ROM) (i.e. the ROM while reaching along the ARM Guide). Targets were located at the limit of the subject's workspace in each of the pre-assigned directions, where this limit was individualized for each subject with the elbow extended and the shoulder flexed as much as possible without pain. For subjects who could not voluntarily move through this entire passive ROM (8 subjects out of 10), the training task was to reach as fast and as far as possible and prescribed trajectories for the active assistance were planned at velocities 20% greater than those that they were able to achieve without assistance. The screening process for this study did not exclude individuals with significant spasticity. While many participants tended to co-contract during volitional movement, none exhibited hyperactive stretch reflex in the range of speeds used for training – namely speeds slightly greater than their maximum voluntary speeds – as confirmed by electromyographical (EMG) recordings during the pre-training evaluations. The choice of training at speeds 20% greater than the maximum voluntary speed was somewhat arbitrary, but was chosen to reinforce movements that were marginally better than their current abilities demonstrated during the eight pre-training reaches at each session. For subjects who could achieve full ROM before training (N = 2), movements were planned by the device at velocities equal to those measured using their ipsilesional arms during unsupported reaches at a self-selected, comfortable speed. Graphical feedback of the amount of assistance provided by the motor was provided after every fifth reach, and subjects were instructed to try to reduce this assistance level. The feedback was used not only to inform subjects of how they were interacting with the device, but also as a motivational factor to encourage improvement of the reaching performance and to keep them intellectually involved in the task.

#### Unassisted free reaching training

Subjects randomized to the free reaching training group performed a matched number of reaches to the same targets as those in the active-assist training group. In this case, the subjects were not attached to the device, and there was no limb support against gravity or any mechanical constraint for arm movement. The initiation point for every movement was with the hand resting on the lap at the umbilicus. All movements were recorded using the Flock of Birds three-dimensional electromagnetic motion capture system (Ascension Technologies, Burlington, Vermont). Subjects were instructed to reach as fast as possible to the target, maintain their position for one second, and then relax. For this task a graph of how close each reach was to the target and a graph indicating the straightness of each movement (described in the Free Reaching Analysis subsection) were provided as feedback after every fifth trial.

### Evaluation

Subjects were evaluated using three exams: a biomechanical examination of the impaired limb with the ARM Guide, a characterization of free reaching, and clinical tests of functional performance. The ARM Guide and free reaching evaluations were repeated once on each subject's ipsilesional arm for normalization.

#### Biomechanical assessment with the ARM guide

The ARM Guide was used to obtain measurements of limb stiffness and supported reaching range and velocity. During slow stretches the load cell recorded the change in resistance force of the passive limb to the stretch as a measure of stiffness [22]. To assess active supported (i.e. with the weight of the arm supported by the device) ROM and maximum velocity, the subjects were instructed to reach as far and as fast as possible along the Guide to target 3 (Figure 1c) without any assistance from the motor. The supported ROM was quantified by calculating the supported fraction of range (FR_S_), defined as the distance traveled by the subject's hand from the starting position, normalized to the same measure for the ipsilesional limb. A score of 1.0 on the supported FR thus indicated that the subject could reach to the full range of motion with the arm supported in the robotic device. Supported reaching speed was normalized to the less affected limb in the same way and referred to as supported fraction of speed (FS_S_). The assessment was performed three times – once on each of three consecutive weeks before the training program began – to identify any baseline trends, and then repeated on three consecutive weeks immediately after training and once at a six month post-training follow up evaluation.

#### Free reaching analysis

The Flock of Birds system was used to capture the path of the hand during three-dimensional unsupported reaching movements to all five targets (see Kamper et al [26] for more details). Additionally, reaches to a target that was not utilized during the training program (transfer target, "Tr" in Figure 1c) were performed to analyze possible transfer of motor recovery in the trained target directions to other areas of the workspace. Unsupported fraction of range (FR_U_) for this task was defined as the linear distance traveled by the subject's hand from the starting position to the closest point to the target and was normalized to the same measure for the ipsilesional limb (contralesional distance/ipsilesional distance). Since subjects were instructed to perform these movements at a comfortable pace, movement speed was not measured and unsupported fraction of speed (FS_U_) is not reported.

The "quality" of unsupported reaching was also assessed during the free reaches using two measures. First, a path length ratio was used as an index of straightness of a reach. It was defined as [26]:



Second, the smoothness of reaching movements was defined by the number of peaks in the hand speed per second. The number of speed peaks measure has been used elsewhere in the literature to describe smoothness [26-28] and has been shown to correlate with other methods for quantifying smoothness, including mean jerk [29]. In this case, since subjects were instructed to move at a comfortable pace with no cues for speed, the measure was divided by movement time to account for slower movements that may have had more peaks solely due to greater movement time. Free reaching measurements were taken on each of two consecutive weeks before the training program began, on two consecutive weeks after training, and one more time at follow-up.

#### Functional assessment

In addition to the Chedoke-McMaster test, the Rancho Los Amigos Functional Test for the hemiparetic upper extremity was used to quantify functional movement ability. This test, performed by a blinded evaluator, consists of a series of timed activities of daily living (ADLs) such as placing a pillow case on a pillow or buttoning a shirt, and it has been shown to have high inter- and intra-rater reliability [30]. The tasks range from simple single joint movements at the shoulder, through simple multijoint movements, to complex multijoint movements involving the hand as well as the arm. To provide finer resolution than the seven-level summary scale (based on pass-fail criteria) developed by the creators of this test, performance was quantified as the mean change in time to completion per task from pre- to post-training. Functional assessments were performed once before the training program and once at its completion.

To summarize, the three different assessments provided eight quantitative outcome measures of arm movement ability: passive stiffness, supported range, supported velocity, unsupported range, unsupported smoothness, unsupported straightness, Chedoke score, and time to complete tasks on the Rancho Los Amigos Functional Test (Table 2).

**Table 2:** Univariate ANOVA statistics for planned comparison of pre- to post-training

	Outcome Measure	Training Group	Mean value before training (SD)	Mean change in value after training (SD)	p value session	p value^† ^session × group	p value^† ^session × impairment
**Biomechanical Evaluation**		Active-assist	0.689 (0.21)	0.139 (0.11)			
	FR_S_				< 0.001*	0.844	0.286
		Free reaching	0.547 (0.14)	0.123 (0.09)			
	
		Active-assist	0.538 (0.14)	0.218 (0.09)			
	FS_S_				< 0.001*	0.898	0.999
		Free reaching	0.430 (0.12)	0.211 (0.13)			
	
		Active-assist	1.114 (0.32)	0.005 (0.25)			
	Stiffness[N/cm]				0.830	0.419	0.006*
		Free reaching	1.400 (0.32)	-0.046 (0.13)			

**Free Reaching Evaluation**		Active-assist	0.768 (0.30)	0.011 (0.09)			
	FR_U_					0.443	0.687	0.710
		Free reaching	0.768 (0.19)	0.024 (0.07)			
	
		Active-assist	1.618 (0.33)	-0.108 (0.18)			
	Straightness ratio				0.033*	0.862	0.204
		Free reaching	1.591 (0.30)	-0.085 (0.25)			
	
		Active-assist	2.189 (0.91)	0.385 (0.62)			
	Smoothness [# speed peaks per sec]				0.128	0.002*	0.086
		Free reaching	2.671 (1.16)	-0.725 (0.72)			

**Functional Evaluation**		Active-assist	3.5 (0.9)	0.2 (0.4)			
	CM Score				0.014*	0.414	0.246
		Free reaching	3.2 (1.0)	0.3 (0.5)			
	
		Active-assist	16.49 (14.5)	-6.28 (11.48)			
				0.048*	0.470	0.438
		Free reaching	10.44 (6.0)	-2.69 (2.02)			

^† ^Session represents evaluation time, with the three pre-training and the three post-training evaluations contrasted in the planned comparison. Training group, session, and impairment level were used in all ANOVAs. Headings with "×" between the factors represent interaction effects.

* p < 0.05

#### Statistical analysis

An initial statistical analysis was made using a doubly multivariate repeated measures analysis of variance (ANOVA), with evaluation session as the within-subject (repeated) factor and treatment group and impairment level as between-subject factors [31]. Separate multivariate ANOVAs were conducted for the biomechanical assessment outcome variables, the free reaching outcome variables, and the functional assessments since each type of evaluation occurred a different number of times. For the purpose of the repeated measures, evaluations were numbered continuously (e.g. the three pre-training biomechanical evaluations were numbered 1 through 3, the three post-training evaluations 4 through 6, and the follow-up 7). ANOVA with the free reaching outcome measures included a second within-subject factor of target, where all six targets were used. In any multivariate ANOVA that exhibited significance, three post-hoc univariate planned comparisons for each outcome variable were used to assess the statistical significance between the following pairs of evaluations: pre- to post-training, pre-training to follow-up, and post-training to follow-up.

**Figure 2:**
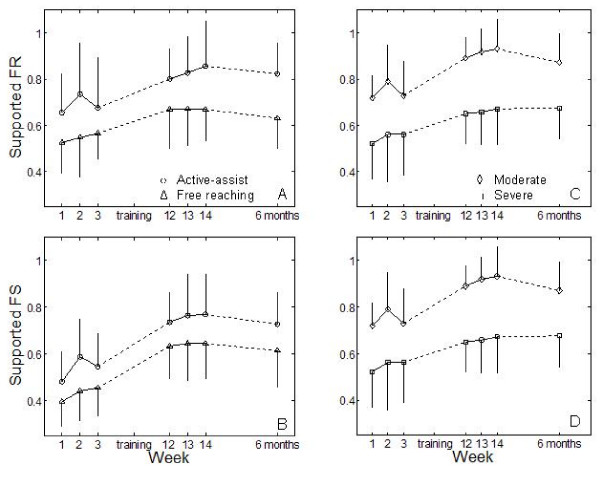
**Changes in supported fraction of range and fraction of speed**. Values are shown for the three preliminary evaluations (weeks 1, 2, and 3), three post-therapy evaluations (weeks 12, 13, and 14), and at the 6-month follow-up evaluation. Plots A and B (left column) show the improved FR_S _and FS_S _after the training period and sustained values at follow-up for participants in both free reaching and active-assist protocols. Plots C and D show the same results for subjects classified by impairment level. Error bars represent standard deviation across subjects. It should be noted that the statistics are designed to detect within-subject differences, while the figures show between-subject means and standard deviations for illustration of mean values.

Apart from minor muscle soreness and fatigue expected of any exercise program, no adverse affects were reported by any participants and nobody withdrew during the training. Two subjects, one in each group, were unable to complete the six-month follow-up because of a recurrent stroke. Since the other subjects grouped together did not show any change from final post-training evaluation to follow-up in the biomechanical measures (p > 0.5), the values at follow-up for the two missing subjects were extrapolated to be equal to their post-training values for graphical representation of means (Figure 2). For the purposes of analyzing recovery as a function of impairment level, subjects with CM scores of 2 and 3 were grouped into a "severely impaired" group, and those with CM scores of 4 or 5 into a "moderately impaired" group.

## Results

At the start of the training program, the subjects exhibited substantial arm movement impairment, and active-assist and free reaching groups were not significantly different from each other for any of the outcome measures. Furthermore, the subjects as a population exhibited a stable baseline during the three pre-evaluations: a comparison of performances of supported reaching during three consecutive weeks before training did not reveal any significant trends (mixed model ANOVA on FR_S _p > 0.72, FS_S _p > 0.24, see weeks 1–3 in Figure 2A,B). Changes following the exercise program for all three sets of evaluations are summarized in Table 2. For the biomechanical evaluation, the doubly multivariate repeated measures ANOVA showed evaluation number to be a significant factor (multivariate p < 0.001), supporting the alternate hypothesis that the outcome measures changed with training. There was, however, no difference in these changes between training groups as evidenced by the lack of interaction effect for session and group (p > 0.85). Differences in changes between severely and moderately impaired groups narrowly missed significance (p = 0.06).

Univariate ANOVA comparing pre- to post-training values showed the improvements in FS_S _and FR_S _for all subjects to be significant (p < 0.001), with no difference in those changes between the training groups (p > 0.8) or impairment levels (p > 0.28) (Figure 2). The only outcome variable from the biomechanical evaluation to not realize a significant change in all subjects was passive limb stiffness, which decreased by 12.7% only in more severely impaired subjects (p < 0.01).

The improvements in the biomechanical outcomes following completion of the training protocol were also present at follow-up (Figure 2). While FR_S _and FS_S _were not different between post-training evaluation and follow up (p = 0.53 and p = 0.81 respectively) there remained a difference from the pre-training values (p < 0.01 for both) based on the univariate planned comparisons. Again, passive stiffness was not significantly different regardless of evaluation time.

For the free reaching evaluation (Table 2), the multivariate ANOVA identified evaluation number (p < 0.03) and target location (p < 0.001) to be significant factors. Furthermore, the combined effect of evaluation number with training group was significant (p < 0.01). Univariate analysis with the planned comparisons revealed that the straightness ratio decreased (i.e. straighter movement) across all subjects after training (p < 0.05). Furthermore, smoothness improved more for the free reaching group as indicated by the interaction of session and training group (p < 0.01). Although reaching performance was different across targets (p < 0.001), there were no differential changes after training (p > 0.3). At the six-month follow-up all changes in unsupported movement kinematics were still present (p < 0.05 comparing pre-training to follow-up, p > 0.23 comparing post-training to follow-up) except that the smoothness improvements in the free reaching group were no longer significant (p > 0.12).

For the functional assessments, there was a significant effect of evaluation time on the functional scores (multivariate p < 0.01, Table 2). The combined effects of evaluation time with training group and evaluation time with impairment level once again were not significant (p > 0.4), indicating that the improvements in functional performance were comparable across treatment groups. In univariate tests, each assessment independently revealed significant improvements with training (p < 0.05), with similarity across treatment groups (p > 0.24) when including the Rancho Los Amigos Assessment as a time-to-completion test. The Rancho Los Amigos Assessment is also designed with a seven-level tiered scoring based on the number of tasks completed with a pass-fail criterion rather than using the continuous scale of the average time-to-completion. Examining the tiered scoring for this assessment, the mean score across groups did not change (pre 4.06 ± 1.75 SD, post 4.18 ± 1.67 SD, p > 0.15) nor was there a difference in the changes between groups (training group × evaluation time p > 0.9). However, three subjects in each group completed at least one additional task after training that they were not able to accomplish before.

**Figure 3:**
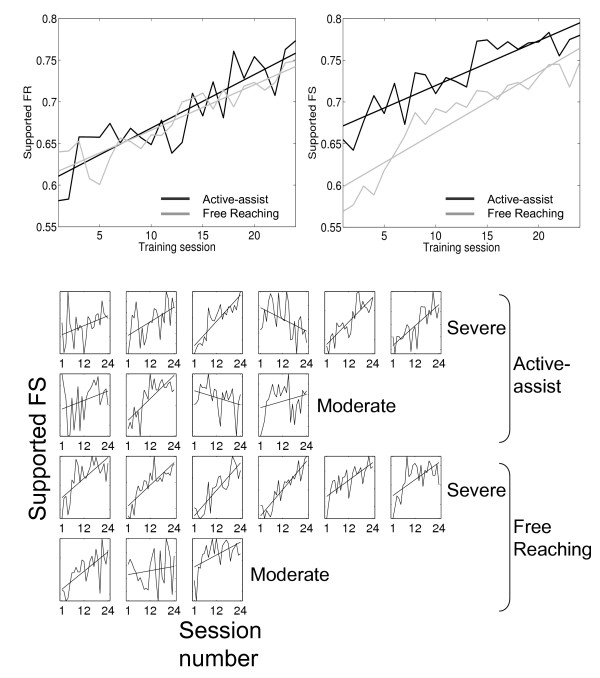
**Mean changes in FR_S _and FS_S _by training session**. The lower array of plots, each representing a single subject, is included to demonstrate that the mean plots are representative of consistent, steady improvements throughout the course of therapy in individual subjects.

To obtain a more detailed picture of the time course of motor improvements, the day-today progress of motor recovery was monitored by measuring eight unassisted, maximum velocity reaches along the robotic device at the beginning of every training session for each subject. The active assistance and free reaching groups both gradually improved their supported reaching range (p < 0.005) and velocity (p < 0.001) at comparable rates (training group × session p > 0.5, Figure 3). Nine of the eleven subjects who began with less than full supported range of motion exhibited significant positive trends in range, while fourteen out of all nineteen subjects showed significant improvements in velocity.

## Discussion

The primary goal of this study was to explore the potential effects of active assistance, delivered by a simple robotic device, in rehabilitation training of the chronic hemiparetic arm. Subjects in both training groups performed equal numbers of reaching movements to identical targets, participated in sessions lasting an equal amount of time, and received graphical feedback of performance throughout each session, but only one group received mechanical assistance that helped complete the desired movement. Both groups significantly improved their range of motion and velocity of supported arm movement, and decreased the time to perform functional tasks. Range of free reaching did not improve with training but straightness did. Participants who practiced free reaching improved the smoothness of their movements. Improvements measured immediately following training were also present at a six month follow-up.

The significant improvements in supported range, supported speed, unsupported straightness, and time to complete functional tasks for both training groups suggest that the repeated attempt to perform the desired movements was a key stimulus for the observed motor recovery. This stimulus of the subject practicing movement, with or without assistance, appears to have had a slow and gradual effect: range and speed improved gradually and continuously over training (Figure 3), at a comparable rate for both training groups, which practiced matched amounts of movements. It is noted that the trends for two individuals presented negative regression slopes in the illustrative plots for each subject in Figure 3. This is not to imply that any participants degraded in their arm movement ability. Rather, this observation is explained by normal variability in the session-to-session changes added onto the potential ceiling effect in subjects whose contralesional limb performance neared their ipsilesional limb performance for this specific measure.

The fact that two participants in the robot-based training performed a slightly different task (sub-maximal speed matching rather than maximum speed) could have potentially confounded these results. However, removal of these two subjects from the analysis had no impact on the outcomes. The multivariate ANOVA on the biomechanical outcomes still indicated improvements post-training in the entire subject pool (p < 0.001) and a lack of differences in those improvements between the two training groups (interaction p > 0.9). The same was true for the univariate tests on FR_S_, FS_S_, and stiffness (p < 0.001, p < 0.001, and p > 0.7 respectively pre- to post-training). Likewise, the univariate and multivariate tests did not change in the free reaching or functional measures indicating that any effect of this variation in training task on the outcomes was negligible.

The only significant difference between the two training groups favored the group that trained with free reaching. The greater improvement in unsupported reaching smoothness by the free reaching group may have been due to the fact that the task being measured in this evaluation was identical to the one that was practiced by this group. Further, the robotic device enforced movements to be smoother in the active-assist trained group; the effect of reducing movement errors may have been to diminish the motor system's attempts to correct those errors. This is in agreement with recent findings comparing the relative effects of trajectory error amplification and error reduction in upper extremity movement practice for individuals with chronic hemiparesis [32].

An interesting finding was that the subjects improved their ability to perform functional tasks, but did not improve the unsupported range of reaching. A possible explanation is that the functional tasks were performed on a table with the objects being manipulated kept close to the body. Free reaching required subjects to attempt to locate the hand away from the body, requiring considerable shoulder strength. The shoulder strength increases caused by the present movement training program may have been substantial enough to improve supported reaching and some proximal movements associated with the functional tasks, but not large enough to improve the ability to extend the arm against gravity.

The finding that the robotic active assistance did not provide statistically significant, additional value beyond the movement practice that occurred concurrently with it should be taken with several caveats. First and foremost, the small sample size of this pilot study precludes estimating the precise difference between the active-assist and free reaching groups. However, we can statistically estimate the maximal likely difference for specific measures. Especially helpful here is the technique of longitudinal power analysis, which uses data from repeated measures to more powerfully estimate the maximal likely difference of those measures [35]. Using this technique in post-hoc analysis, there was an 80% probability that the statistics would have detected a 30% difference in the FS_S _(the most consistent outcome measure, and thus that associated with the greatest power), which was measured at each training session. Thus, if there was a difference between active-assist and free reaching exercise in affecting maximum speed of reaching, for example, it was likely incremental rather than dramatic.

A second caveat is that the particular active-assist technique implemented here may not have been optimal. The active-assist algorithm that we used had the following features: it required the subject to initiate movement, completed abnormal movements along a suitable trajectory, and provided graphical feedback of the subject's contribution to movement. However, a therapist, or a better-designed robotic movement training system, may be better able to discern when exactly assistance is needed, and may be better able to grade the level of assistance needed. In fact, comparisons of therapeutic approaches incorporating some form of clinician assistance have revealed differing rates of motor recovery and cortical reorganization in a subacute population [36] and specially designed adaptive robotic therapy has been hypothesized to stimulate greater recovery in a chronic population[37]. Progressively reducing the amount of assistance throughout training may also promote motor learning [38,39].

A third caveat in assessing the role of the robotic assistance per se in motor rehabilitation is that the subject population was diverse in terms of impairment level and lesion location. It may be that active-assist training will eventually be determined to be beneficial for specific subgroups of patients, such as those with proprioceptive deficits, high levels of spasticity, or perhaps during acute recovery for flaccid patients. Much of the stroke rehabilitation literature is divided by the stage of recovery of study participants, with some studies concentrating on subacute stroke and others focusing on chronic stroke. The more rapid rates of recovery in individuals with subacute stroke who trained with the robot (as compared to the control group) in the initial MIT-MANUS study [13] raise the possibility that the same active-assistance used here could be more potent as an earlier intervention. Similarly, intensification of a training program can magnify the effects at any stage of recovery [40]. While the possibility still exists for the outcomes for the two training methods used in this study to be different depending on a number of parameters, such differences at this point are still speculative and require future study.

The findings of this study confirm again the potential for robotic devices to elicit improved upper extremity movement ability in individuals with chronic hemiparesis following a stroke. However, they suggest that caution is warranted in interpreting the effects from exercise with such devices when they are used to assist in movement training: while not detrimental, the device in the present study did not provide any detectable value beyond that achievable with a matched amount of practice and no assistance. Despite this, there are many possible robotic movement training techniques, and we tested only one. There is some preliminary evidence that alternate forms of robot therapy that focus on directing the patient's effort may be more effective at improving unsupported movement range than the active-assist approach selected in the present study [37,41]. Thus, while repetitive movement practice should likely comprise the core of treatment for advancing arm recovery after stroke, addition of other compatible approaches may further enhance specific aspects of movement ability.

## Conclusion

The significant improvements in movement ability found with both active-assist and unassisted training further supports repetitive movement training as a viable strategy for improving impairment of the hemiparetic upper extremity after chronic stroke, with or without a robotic device. The robotic assistance incorporated here did not provide any detectable benefits beyond the unassisted movement exercise, though interpretation of this result is limited by sample size and to the specific assistance technique employed with the device. Changes in motor function for both training strategies were gradual and the possibility remains that other unique interactions with robotic devices may be designed for patients with specific types of stroke and at specific stages of recovery to amplify the effects seen here with simple active-assistance.

## Competing interests

The author(s) declare that they have no competing interests.

## Authors' contributions

LK and MZ were involved in all stages of subject recruitment and data acquisition. LK was also the primary composer of the manuscript. DR and ZR generated the initial concept for the study and oversaw its progress. DR also designed and built the robotic device used for training. All four authors contributed significantly to the intellectual content of the manuscript and have given final approval of the version to be published.
